# Valuation of Investments in Hospital Accounts

**Published:** 1913-06-21

**Authors:** Alfred M. Hooper


### VALUATION OF INVESTMENTS IN HOSPITAL ACCOUNTS.

### A Suggestion for Securing Uniformity.

By ALFRED M. HOOPER.

The Uniform System of Accounts adopted by King Edward's and the "Sunday" and "Saturday" Funds was devised, primarily, to facilitate truer comparison than had hitherto been possible between the expenditure of various hospitals; but it was doubtless also intended to be a means of ascertaining their relative financial position with a view to gauging to what extent each was in need of assistance.

While this system regulates the form in which the final accounts are presented to the public, the methods by which the results are arrived at may vary considerably. In the special form of balance-sheet, annotated for the guidance of hospital officials (*vide* Red-book 1909, page 39, paragraph 5 c), it is laid down, under investments on capital accounts, referring to stock:

"*It is desirable to state the investments at cost price except in cases where important depreciation of what is believed to be a permanent character has happened, when the cost price may be written down and the security stated at a lower value.*"

This seems to leave the valuation of these assets much to individual discretion, and it has evidently been generally interpreted as an invitation to value at cost. An examination of a number of the balance-sheets published by hospitals shows that in the majority of cases this has been done. In some instances a valuation has been made at a given year and subsequent purchases taken at cost; in others, values are placed on stock without any indication as to how or why they are so valued.

### Misrepresentation of Assets.

The result, as one would naturally expect owing to the great depreciation of high-class stocks that has been taking place for some years, is that in nearly every case the values entered on the balance-sheet are greatly in excess of the amounts that could be expected by sale. In one case a sum of £27,000 stands as an asset, while a footnote explains that the actual value is about £4,500 less.

While the various hospitals compile their accounts on such diverse, and I think it may be said on such unsatisfactory, methods, the real position of their finances cannot be accurately ascertained without considerably more investigation than the public is likely to make. It is difficult to understand why hospital authorities should be content to allow their assets to be overestimated and thus misrepresent their position; probably it is due to the want of more definite guidance on the point by the three big "Funds."

It seems desirable, if only for the sake of aiding comparison, that some more accurate basis of valuation should be arrived at and generally adopted. It may be contended that it is impossible to fix a value that is reliable for any length of time for securities that fluctuate in price, but there seems no reason why this should prevent an endeavour to find a more satisfactory method of valuation nor indeed why every hospital should not employ the same principles. If securities were to be shown in the balance-sheets of all hospitals at their value in the market at the same date, as, for instance, December 31, and a note added stating this to be the case, comparison between the various institutions would be facilitated and a truer statement of their finances presented. Such yearly valuation would of course necessitate some additional labour and bookkeeping, but no hospital official will grudge the time spent provided it is for the benefit of his institution.

### The Stock Ledger.

It may be of interest to some of the junior officials of hospitals where annual valuations have not been the practice to give some details of the necessary accountancy. In the first place I should like to explain the ruling I advocate for the stock ledger, which is the book chiefly concerned. I advise the keeping of the stock and dividend transactions entirely separate, and for this purpose one page is divided into debit and credit sides for stock and the opposite page similarly for dividends. The stock page is divided on both the Dr. and Cr. sides into columns for date, details, folio, price, nominal amount of stock, and book value. The dividend sides into date, particulars, folio, and double cash columns for the amounts posted and totals.

Dealing with the stock transactions first, we shall have in the price column the rate at which the stock was last valued, in the value column the value of the stock at such price, and of course in the stock column the nominal value of the stock. In arriving at the amount of depreciation or appreciation at the date at which it is desired to re-value, it will be necessary to take into consideration whether the rate taken is ex div. or cum div.; in the former case the gross amount is taken, but in the latter such proportion of the dividend as is due at the date of valuation will have to be deducted. The subsequent transactions are merely those of writing off on depreciation or appreciation respectively and carrying forward the balances.

It will be seen that where the amount of stock held is small not much extra labour is involved; even if a considerable time has to be expended it is surely of importance that the financial position of the institution should be presented to the public as correctly as possible.

